# Predicting the Progress of Caustic Injury to Complicated Gastric Outlet Obstruction and Esophageal Stricture, Using Modified Endoscopic Mucosal Injury Grading Scale

**DOI:** 10.1155/2014/919870

**Published:** 2014-08-04

**Authors:** Lung-Sheng Lu, Wei-Chen Tai, Ming-Luen Hu, Keng-Liang Wu, Yi-Chun Chiu

**Affiliations:** Division of Hepato-Gastroenterology, Department of Internal Medicine, Kaohsiung Chang Gung Memorial Hospital and Chang Gung University, College of Medicine, 123 Ta-Pei Road, Niao-Sung Hsiang, Kaohsiung 833, Taiwan

## Abstract

Severe caustic injury to the gastrointestinal tract carries a high risk of luminal strictures. The aim of this retrospective study was to identify predicting factors for progress of caustic injury to gastric outlet obstruction (GOO) and esophageal strictures (ES), using modified endoscopic mucosal injury grading scale. We retrospectively reviewed medical records of patients with caustic injuries to the gastrointestinal tract in our hospital in the past 7 years. We enrolled 108 patients (49 male, 59 female, mean age 50.1 years, range 18–86) after applying strict exclusion criteria. All patients received early upper gastrointestinal endoscopy within 24 hours of ingestion. Grade III stomach injuries were found in 58 patients (53.7%); 43 (39.8%) esophageal, and 13 (12%) duodenal. Of the 108 patients, 10 (9.3%) died during the acute stage. Age over 60 years (OR 4.725, *P* = 0.029) was an independent risk factor of mortality for patients after corrosive injury. Among the 98 survivors, 36 developed luminal strictures (37.1%): ES in 18 patients (18.6%), GOO in 7 (7.2%), and both ES and GOO in 11 (11.3%). Grade III esophageal (OR 3.079, *P* = 0.039) or stomach (OR 18.972, *P* = 0.007) injuries were independent risk factors for obstructions. Age ≥60 years was the independent risk factor for mortality after corrosive injury of GI tract. Grade III injury of esophagus was the independent risk factor for development of ES. Grade III injury of stomach was the independent risk factor for development of GOO.

## 1. Introduction

Patients who have experienced severe caustic injury to the gastrointestinal (GI) tract are at high risk of luminal strictures [1]. Early endoscopy is usually routinely recommended in patients after gastroesophageal caustic injuries and should be performed to prevent unnecessary hospitalization and to plan future treatment after carefully assessing the severity of the initial digestive lesions [2]. Ingested corrosive substances are either alkalis or acids, and these produce different types of tissue damage [3]. Acids cause coagulation necrosis, whereas alkalis combine with tissue proteins to liquefy and cause necrosis to penetrate deep into tissues [4]. Alkali ingestion may lead to more serious injury and complications by penetrating tissues and leading to full-thickness damage of the esophageal/gastric wall [5].

Gastroesophageal strictures can be severely injurious for patients who survive caustic injuries. The incidence of esophageal stricture (ES) following grade IIB and grade III esophageal burns is very high, at 70–100% [6, 7]. Gastric outlet obstruction can be a late sequel to caustic stomach injury, mainly in the prepyloric area, causing gastric outlet obstruction (GOO). The reported incidence of GOO caused by caustic stomach injury is more than 60% [3, 8]. Timely evaluation and endoscopic pneumatic dilations of the stricture have been widely practiced and reported in the literature and are now one of the standard treatment options; however, different outcomes have been reported for patients with ES alone, GOO alone, or a combination of both ES and GOO [9–13]. Therefore, it is important to identify which patients could potentially progress to ES or GOO.

The aim of this retrospective study was to identify the predicting factors for the progress of caustic injury to GOO and ES, using MEMIGS.

## 2. Patients and Methods

### 2.1. Ethics Approval

This study was approved by both the Institutional Review Board and Ethics Committee of Chang Gung Memorial Hospital.

### 2.2. Patients

We retrospectively reviewed the medical records of patients who had ingested a corrosive substance and were admitted to our hospital between July 2003 and December 2010. These patients had received early upper GI endoscopy (GIF-Q240; Olympus Optical Co., Ltd., Tokyo, Japan) within 48 hours of ingestion. During endoscopy, air insufflation and retroflexion were carefully performed to avoid iatrogenic injury. Endoscopy was performed in all patients without sedation to minimize the risk of aspiration. Mucosal burns of the esophagus, stomach, and duodenum were graded by modifying the method previously reported by Zargar et al.: grade 0, normal examination; grade I, edema and hyperemia of the mucosa; grade II, subdivided into grade IIa (friability, hemorrhages, erosions, blisters, whitish membranes, exudates, and superficial ulcerations); grade IIb (grade IIa plus deep discrete or circumferential ulceration); and grade III, multiple ulcerations and areas of necrosis [5]. Esophageal, gastric, and duodenal injuries were graded separately. Patients with grade I and IIa burns were discharged after endoscopy, while those with grade IIb and burns remained hospitalized.

During admission, patients were treated with a proton pump inhibitor or H2 antagonist and were maintained without oral intake until their condition was considered stable. Patients received parenteral nutrition during this period. If infection was suspected, antibiotics (first-generation antibiotics, such as cephalosporin and gentamicin) were administered after blood cultures were obtained. If a patient's condition destabilized or respiratory difficulty was encountered, they were transferred to the intensive care unit for further evaluation. If the patient demonstrated symptoms of upper GI stricture, including dysphagia, easy satiety, or postprandial vomiting, endoscopy was performed in the fourth week after the corrosive injury to examine the upper GI tract. Stricture was defined as dysphagia, symptoms of regurgitation, or difficulty in swallowing, with confirmation by endoscopy and/or upper GI radiography. After discharge, patients were followed up in the outpatient clinic for at least 6 months. Exclusion criteria were (1) patients who died during the acute stage of corrosive injury and (2) patients who were unable to give informed consent for endoscopic balloon dilation (EBD).

Clinical information, including the amount and type of ingested corrosive substance, hospital course, and complications, was collected. Body weight at time of presentation and follow-up was also recorded. Presence and severity of difficulty in swallowing and/or postprandial satiety and vomiting in the follow-up period were recorded at each clinical follow-up.

### 2.3. Study End-Point

The end-point was the development of GOO and ES during endoscopic follow-up.

### 2.4. Statistical Analysis

Continuous variables are given as means and standard deviation. The continuous variables were analyzed by the Mann-Whitney* U*-test. Categorical variables are given as total and percentages and were analyzed using the *χ* ^2^ tests or Fisher's exact test. Univariate and multivariate logistic regression were used to analyze the factors related to mortality and stricture development after corrosive injury. Two-sided *P* value of <0.05 was considered significant. All statistical operations were performed using SPSS (Statistical Package for the Social Sciences version 15, Chicago, IL, USA).

## 3. Results

A total of 108 medical records of patients (49 male, 59 female, mean age 50.1 years, range 18–86) with diagnosis of caustic GI tract injury were reviewed. The corrosive substance ingested was acid in 90 cases and alkali in 18.  Table 1 shows the degree and extent of their injuries based on the results of endoscopic examination performed within 48 hours of ingestion. Grade III corrosive injury was noted in the esophagus in 43 (39.8%) patients, in the stomach in 58 (53.7%), and in the duodenum in 13 (12%). Tables 2 and 3 show the clinical characteristics and severity of corrosive injury in patients with and without ES and GOO. Patients with ES had more severe (at least grade III) injuries to the esophagus (58.6% versus 27.5%, *P* = 0.006) and stomach (65.5% versus 43.5%, *P* = 0.046) than those without ES. Patients who developed GOO had more severe injuries to the stomach (100% versus 38.8%, *P* < 0.001) and duodenum (43.8% versus 8%, *P* < 0.001) than those without GOO.

Of the 108 patients, 10 (9.3%) died during the acute stage as a result of esophageal perforation (*n* = 2), hematemesis with sudden apnea (*n* = 1) or aspiration pneumonia with respiratory failure (*n* = 7). The average duration of hospital stay was 11.1 ± 7.6 days, with an intensive care unit (ICU) admission rate of 15.6%.

As shown in Table 4, univariate analysis of mortality rate demonstrated that age of over 60 years (O.R 6.636, *P* = 0.004) and grade III injury to the stomach (O.R 5.106, *P* = 0.042) correlated with high mortality rates. Multivariate analysis revealed that only age over 60 years (O.R 4.725, *P* = 0.029) was an independent risk factor of mortality for patients after corrosive injury.

The remaining 98 (90.7%) of the 108 patients who survived the acute stage of corrosive injury were enrolled. Of these survivors, 36 (36.7%) developed intake problems. ES alone was found in 18 patients (18.4%), GOO alone in 7 (7.1%), and a combination of ES and GOO in 11 (11.2%). The overall incidence of ES and GOO was 29.6% (29/98) and 18.4% (18/98), respectively. In ES group, patients received a total of 110 sessions of endoscopic balloon dilation (EBD) with an average of 6.1 + 4.7 sessions per patient. In GOO group, patients received a total of 39 sessions of EBD with an average of 5.5 + 2.1. In ES + GOO group, patients received 152 sessions of EBD with an average 13.8 ± 4.9. The success rates to achieve symptom relief were 83.3% (15/18) in ES group, 57.1% (4/7) in GOO group, and 36.4% (4/11) in ES + GOO group. The rest of the patients with unsuccessful EBD underwent surgical treatment with success. No mortality was related to EBD and surgical treatment.

Univariate and multivariate analyses of ES and GOO by logistic regression are shown in Tables 5 and 6. Univariate analysis of ES demonstrated that patients with grade III injury to the esophagus (OR 3.728, *P* = 0.005) or stomach (OR 2.470, *P* = 0.049) had a significantly higher incidence of ES than those without such extensive injury. Multivariate analysis revealed that only grade III injury to the esophagus (OR 3.079, *P* = 0.039) was an independent risk factor of ES for patients after corrosive injury. Univariate analysis of GOO demonstrated that patients with grade III injury to the stomach (OR 33.103, *P* = 0.001) and the duodenum (OR 10.182, *P* < 0.001) had a significantly higher incidence of GOO than those without such extensive injury. Multivariate analysis revealed that only grade III injury to the stomach (OR 18.972, *P* = 0.007) was an independent risk factor of GOO for patients after corrosive injury.

## 4. Discussion

The incidence of corrosive ingestion is high and largely unreported in developing countries, where prevention is lacking [3]. It is a serious public health concern worldwide [3]. The acute stage of treatment is very important and in many cases, the results of such ingestion can be very serious. The need to perform emergency surgery has a persistent long-term negative impact both on survival and functional outcome [3]. In the current study, 10 patients (9.3%) died during the acute stage as a result of esophageal perforation (*n* = 2), hematemesis with sudden apnea (*n* = 1), or aspiration pneumonia with respiratory failure (*n* = 7). The remaining 98 (89.8%) of the 108 patients survived the acute stage of corrosive injury. The average duration of hospital stay was 11.1 ± 7.6 days with an ICU admission rate of 15.6%.

However, for those who survive the acute stage, the late complications of corrosive gastric injury include intractable pain, gastric outlet obstruction, late achlorhydria, protein-losing gastroenteropathy, mucosal metaplasia, and development of carcinoma [3, 14]. In this study, we focused on the late complication of obstructions, such as GOO and ES. EBD remains the most important treatment option for luminal stricture caused by caustic injuries [9–12], and although it has been reported to be generally safe and effective, serious complications may occur, especially when there is concomitant existence of GOO and ES [13]. Therefore, identification of clinical factors to predict the possible occurrences of GOO and ES is an important issue. In the current study, we used endoscopic parameters in an attempt to identify these predictors using modified endoscopic parameters. Our results showed that grade III injury to the stomach and esophagus were independent risk factors of GOO and ES, respectively, for patients after corrosive injury.

Some literatures also found that grade 2b was also related to subsequent luminal strictures [15]. Among the 98 survived patients in our study, the incidence rates of ES were 26.7% (12/45) in grade 2b injury to esophagus and 48.6% (17/35) in grade 3 injury (*P* = 0.043). And the incidence rates of GOO were 5% (1/20) in grade 2b injury to stomach and 34.7% (17/49) in grade 3 injury (*P* = 0.011). No matter in esophagus or stomach, patients with grade 3 injury had a higher risk to develop lumen stricture than those with grade 2b injury.

The relative extent of esophageal and gastric involvement largely depends on the nature and volume of the corrosive ingested [16]. Acids are more likely than alkalis to affect the stomach [17]. Alkalis cause liquefaction necrosis, and as they are more viscous, most of the liquid adheres to the esophageal mucosa, with only a relatively small amount reaching the stomach [18]. Therefore, the extent of esophageal damage is greater with alkalis than with acids. In the current study, the corrosive substance ingested was acid in 90 cases and alkali in 18. None of the 18 patients with GOO alone or with concomitant GOO and ES had ingested alkaline substances. Thus, a possible explanation for progress to GOO could be the prolonged contact of corrosive agents with the antral mucosa, as a result of pyloric spasm, causing mucosal damage by coagulation necrosis [3, 19]. Sometimes, in cases of a large volume of corrosive agent being ingested, the damage may be so severe that strictures can be found in the antrum, body, or pyloroduodenal area, so that the entire stomach might be diffusely scarred [18]. As shown in Table 1, we refrained from forcing the scope through the pylorus in 30 cases because of the severe gastric damage. We also observed that all of the patients who developed GOO had at least grade III caustic injuries, compared with slightly over a third of who did not (100% versus 38.8%, *P* < 0.001). Moreover, this was further confirmed by multivariate analysis, showing that severe caustic injury to the stomach of at least grade III was an independent risk factor.

Endoscopy should be avoided within 2 weeks after EBD because of the high risk of perforation, although there is no good evidence in the literature to suggest the best timing to perform this technique [20]. However it is known that EBD can be performed effectively and safely 4–6 weeks after corrosive injury and is the treatment of choice for most of these injuries [21, 22]. Surgery is carried out only in cases with severe complications, when EBD fails or when patients are unable to tolerate EBD procedures. This is because esophagectomy followed by reconstruction surgery is a laborious and invasive procedure that exposes patients to high risks of morbidity and mortality. The same appears to the surgical intervention for GOO, which usually involves subtotal gastrectomy or bypass gastrojejunostomy, although this tends to have fewer complications [23, 24]. Therefore, it is important to identify which patients might potentially progress to ES or GOO. The current study confirmed that the modified endoscopic parameter helps to assess and identify those patients with grade III caustic injuries, allowing physicians to ensure close follow-up and to instigate prompt therapy without delay. A recent study by Cheng et al. also showed that patients with grade III b burns identified by endoscopy have high rates of morbidity. The grading scale by Zargar et al. is useful for predicting immediate and long-term complications and guiding appropriate therapy [15].

Mortality can occur in patients with extensive injuries to both the stomach and the esophagus. In the current study, 10 of the 108 patients (9.3%) died during the acute stage and all of them had severe caustic injuries of at least grade III diagnosed by endoscopic examinations, and all were aged over 60 years. The average duration of hospital stay for patients was 11.1 ± 7.6 days, with an ICU admission rate of 15.6%. However, this could be an underestimate because some patients with severe acute and chronic gastric and esophageal injuries die in peripheral centers before they make it to a tertiary care referral hospital.

## 5. Conclusion

The results of this study indicate that patients over 60 years have a higher mortality rate after corrosive injury of GI tract and, therefore, require attentive care in acute stage. And, early endoscopy to grade the extent of mucosal injury is useful to predict the incidence of subsequent stricture of GI tract and provide valuable information on clinical follow-up.

## Figures and Tables

**Table 1:** Corrosive injury of gastrointestinal tract based on modified endoscopic mucosal injury grading scale.

	Severity of corrosive injury				
	0	I	IIa	IIb	III
Esophagus (*n* = 108)	1	9	9	46	43
Stomach (*n* = 108)	3	13	13	21	58
Duodenum∗ (*n* = 78)	21	24	8	12	13

*Operators refrained from forcing the scope through the pylorus in 30 cases because of severe gastric damage.

**Table 2:** Comparison of the clinical characteristics and severity of corrosive injury between patients with and without esophageal stricture (ES).

	ES *N* = 29	Non-ES *N* = 69	*P*
Age (years, mean ± SD)	45.1 ± 15	49.5 ± 17	0.321
Gender (M/F)	12/17	34/35	0.475
Acid/alkali	25/4	61/8	0.745
Grade III esophagus injury	17 (58.6%)	19 (27.5%)	0.006
Grade III stomach injury	19 (65.5%)	30 (43.5%)	0.046
Grade III duodenum injury	6 (24%)	7 (10.6%)	0.103

**Table 3:** Comparison of the clinical characteristics and severity of corrosive injury between patients with and without gastric outlet obstruction (GOO).

	GOO *N* = 18	Non-GOO *N* = 80	*P*
Age (years, mean ± SD)	50.9 ± 14.9	47.6 ± 17.8	0.445
Gender (M/F)	8/10	38/42	0.814
Acid/alkali	17/1	69/11	0.457
Grade III esophagus injury	9 (50%)	27 (33.8%)	0.279
Grade III stomach injury	18 (100%)	31 (38.8%)	<0.001
Grade III duodenum injury	7 (43.8%)	6 (8%)	<0.001

**Table 4:** Univariate and multivariate analyses of mortality for individual parameters in patients with corrosive injury of gastrointestinal tract.

Parameter∗	Univariate			Multivariate		
	Risk	95% CI	*P*	Risk	95% CI	*P*
Age	6.636	1.824–24.143	0.004	4.725	1.168–19.112	0.029
Gender	0.355	0.090–1.393	0.138	0.228	0.040–1.305	0.097
Acid/alkali	1.440	0.169–12.261	0.738			
Grade of esophagus injury	3.429	0.962–12.215	0.057	2.320	0.468–11.503	0.303
Grade of stomach injury	5.106	1.062–24.558	0.042	3.663	0.592–22.670	0.163
Grade of duodenum injury	3.429	0.879–13.379	0.076	1.104	0.189–6.442	0.913

*Cut-off: age: ≥60 or <60 years; gender: male or female; type of ingestion substance: acid or alkali; grade of esophagus injury: grade III or not; grade of stomach injury: grade III or not; grade of duodenum injury: grade III or not.

**Table 5:** Univariate and multivariate analyses of esophageal stricture for individual parameters in patients with corrosive injury of gastrointestinal tract.

Parameter∗	Univariate			Multivariate		
	O.R	95% CI	*P*	O.R	95% CI	*P*
Age	0.421	0.129–1.371	0.151	0.309	0.078–1.224	0.094
Gender	0.727	0.302–1.746	0.475			
Acid/alkali	0.762	0.226–2.970	0.820			
Grade of esophagus injury	3.728	1.503–9.246	0.005	3.079	1.059–8.948	0.039
Grade of stomach injury	2.470	1.003–6.085	0.049	1.973	0.613–5.969	0.264
Grade of duodenum injury	2.797	0.838–2.887	0.094	1.306	0.324–5.259	0.707

*Cut-off: age: ≥60 or <60 years; gender: male or female; type of ingestion substance: acid or alkali; grade of esophagus injury: grade III or not; grade of stomach injury: grade III or not; grade of duodenum injury: grade III or not.

**Table 6:** Univariate and multivariate analyses of gastric outlet obstruction for individual parameters in patients with corrosive injury of gastrointestinal tract.

Parameter∗	Univariate			Multivariate		
	O.R	95% CI	*P*	O.R	95% CI	*P*
Age	1.024	0.329–3.185	0.967			
Gender	1.305	0.394–2.721	0.944			
Acid/alkali	2.333	0.405–27.422	0.263	1.111	0.105–11.772	0.931
Grade of esophagus injury	2.944	1.094–7.922	0.320			
Grade of stomach injury	33.103	4.217–259.882	0.001	18.972	2.226–158.875	0.007
Grade of duodenum injury	10.182	2.815–36.863	<0.001	3.805	0.983–14.735	0.053

*Cut-off: age: ≥60 or <60 years; gender: male or female; type of ingestion substance: acid or alkali; grade of esophagus injury: grade III or not; grade of stomach injury: grade III or not; grade of duodenum injury: grade III or not.
